# Temperature-Responsive Hydrogel-Coated Gold Nanoshells

**DOI:** 10.3390/gels4020028

**Published:** 2018-03-26

**Authors:** Hye Hun Park, La-ongnuan Srisombat, Andrew C. Jamison, Tingting Liu, Maria D. Marquez, Hansoo Park, Sungbae Lee, Tai-Chou Lee, T. Randall Lee

**Affiliations:** 1 Department of Chemistry and the Texas Center for Superconductivity, University of Houston, Houston, TX 77204-5003, USA; hhpark95@gmail.com (H.H.P.); slaongnuan@yahoo.com (L.S.); andrewcjamison@yahoo.com (A.C.J.); sdzbltt@gmail.com (T.L.); mdmarqu2@gmail.com (M.D.M.); 2 School of Integrative Engineering, Chung-Ang University, Seoul 156-756, Korea; heyshoo@cau.ac.kr; 3 Departments of Physics and Photon Science, Gwangju Institute of Science and Technology, 123 Chemdan-gwagiro (Oryong-dong), Buk-gu, Gwangju 500-712, Korea; jaylinlee@gist.ac.kr; 4 Department of Chemical and Materials Engineering, National Central University, 300 Jhongda Road, Jhongli City 32001, Taiwan; taichoulee@ncu.edu.tw

**Keywords:** drug delivery, temperature responsive, gold nanoshell, hydrogel coating

## Abstract

Gold nanoshells (~160 nm in diameter) were encapsulated within a shell of temperature-responsive poly(*N*-isopropylacrylamide-*co*-acrylic acid) (P(NIPAM-*co*-AA)) using a surface-bound rationally-designed free radical initiator in water for the development of a photothermally-induced drug-delivery system. The morphologies of the resultant hydrogel-coated nanoshells were analyzed by scanning electron microscopy (SEM), while the temperature-responsive behavior of the nanoparticles was characterized by dynamic light scattering (DLS). The diameter of the P(NIPAM-*co*-AA) encapsulated nanoshells decreased as the solution temperature was increased, indicating a collapse of the hydrogel layer with increasing temperatures. In addition, the optical properties of the composite nanoshells were studied by UV-visible spectroscopy. The surface plasmon resonance (SPR) peak of the hydrogel-coated nanoshells appeared at ~800 nm, which lies within the tissue-transparent range that is important for biomedical applications. Furthermore, the periphery of the particles was conjugated with the model protein avidin to modify the hydrogel-coated nanoshells with a fluorescent-tagged biotin, biotin-4-fluorescein (biotin-4-FITC), for colorimetric imaging/monitoring.

## 1. Introduction

A major goal of nanotechnology has been the development of nanoscale materials with functional properties. To this end, gold nanoparticles (AuNPs) are particularly attractive for use in medicinal applications due to their biocompatibility [1] and optical properties, especially their surface plasmon resonance (SPR) [2]. SPR-based biosensors are a popular tool for the detection of biomolecules. For example, a Cy5.5-substrated/AuNP system was reported as a multi-quenched near-infrared-fluorescence probe, providing a visual means to monitor a target protease and its inhibitor [3]. Additionally, AuNPs have been used as sensors for the colorimetric detection of the serum protein homocystamide, a biomarker that aids in the diagnosis of cardiovascular disease [4]. Furthermore, in a recent report, an aptazyme-AuNP sensor was developed as the first example of a sensor that allows for the amplified detection of biomolecules inside living cells [5].

Significant biomedical applications can be realized in vivo when AuNP resonances are tuned to the near infrared (NIR) region by changing the particle shape into hollow structures [6,7,8], gold nanorods [9], or gold nanoshells [10]. Among the different types of AuNPs, gold-silica nanoparticles, which consist of a silica core surrounded by a thin gold layer, have been well investigated fundamentally, as well as their use in a wide range of technically important applications [11,12,13]. In contrast to solid gold nanoparticles, the plasmon resonance of nanoshells can be precisely and systemically varied to specific wavelengths ranging from the UV-Vis to the near IR (NIR) region of the spectrum, by simply adjusting the size of the dielectric core and particularly the thickness of the gold layer [14]. Monodisperse silica nanoparticle cores can be synthesized over extremely broad size ranges, with the core providing one method of tuning the optical properties of the completed nanoshells [14,15]. If the shell thickness is varied on a silica core of constant size, the resulting optical properties are shown to shift as a function of shell thickness; specifically, as the thickness of the gold layer is decreased, the SPR is shifted to longer wavelengths. Absorption in the NIR region, generated through these methods, becomes particularly relevant for biomedical applications mainly due to the transparency of both blood and human tissue in that region of the spectrum, a span of wavelengths called the “phototherapeutic window”; light emitted at wavelengths between 800 and 1200 nm can pass through human tissue and then be absorbed/scattered by the nanoshells [16]. As NIR light interacts with the nanoshells, the radiation is converted efficiently into heat due to electron-phonon and phonon-phonon processes, providing rapid and efficient photothermal heating to the medium surrounding the nanoshells [17]. Using these features, nanoshells have been utilized in photothermal cancer therapy in vitro and in vivo [18]. The nanoshells accumulated at the tumor deliver a therapeutic dose of heat to the cancer cells upon irradiation with NIR light, thereby giving rise to localized photothermal ablation of the tumor tissue. 

Hydrogels are three-dimensional, cross-linked polymer networks composed of more than 90% water. Due to the high porosity of the hydrogel and its ability to swell in aqueous environments, hydrogels play an important role in biological applications. For example, novel inverse opal hydrogel particles were recently developed as enzymatic carriers, making them viable for biocatalytic applications [19]. Notably, a reversible glucose sensor has also been developed, which can sense in the millimolar range via a combination of gold nanoantennas and a boronic acid-functionalized hydrogel [20]. Similarly, phenylboronic acids embedded in a hydrogel network have been reported for glucose sensing [21]. Moreover, a unique technology that embeds nanoparticle-stabilized liposomes into a hydrogel shows promising results toward topical antimicrobial delivery [22].

**Scheme 1:**
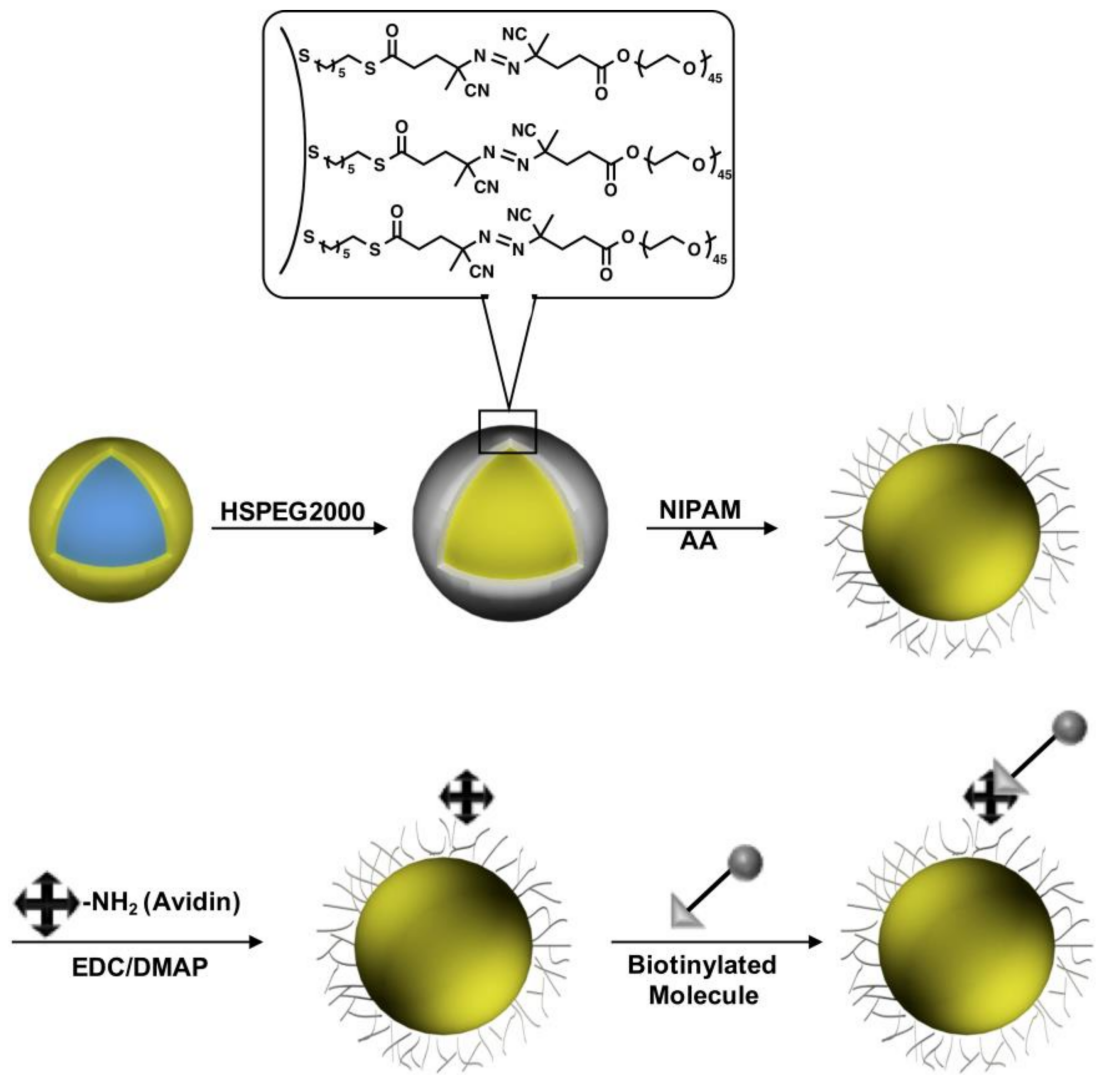
Schematic diagram of hydrogel growth on gold nanoshells using radical polymerization followed by modification of the hydrogel periphery with avidin and biotin-4-FITC molecules. **HSPEG2000**, (*E*)-PEG2000-4-cyano-4-((*E*)-((*R*)-2-cyano-5-(6-mercaptohexylthio)-5-oxopentan-2-yl)diazenyl)pentanoic acid; NIPAM, *N*-isopropylacrylamide; AA, acrylic acid; EDC, *N*-(3-dimethylaminopropyl)-*N*′-ethylcarbodiimide hydrochloride; DMAP, 4-dimethylaminopyridine.

In this current study, gold nanoshells were incorporated into temperature-responsive poly(*N*-isopropylacrylamide-*co*-acrylic acid) (P(NIPAM-*co*-AA)) hydrogels for the development of a multifunctional material with potential use as a photothermally-modulated drug-delivery system (Scheme 1). To afford enhanced structural and compositional control, the hydrogel layer was grown using surface-immobilized radical initiators anchored onto the nanoshells through covalent S-Au bonds. Furthermore, the terminal PEG moieties were added to provide colloidal stability and biocompatibility to the composite nanoparticle system. *N*-isopropylacrylamide (NIPAM)-based hydrogels have been thoroughly studied due to their unique thermal behavior in aqueous solution [23,24,25,26,27,28]. At low temperatures, the poly(*N*-isopropylacrylamide) (PNIPAM) hydrogel exhibits hydrophilic behavior in water, due to hydrogen bonding between the PNIPAM functional groups and water, which causes the material to swell. However, at higher temperatures, there is an entropically-favored release of water molecules embedded in the hydrogel structure that leads to the collapse of the structure. The temperature at which the phase change occurs is called the lower critical solution temperature (LCST), and PNIPAM shows a LCST at 32 °C [29]. Introducing acrylic acid (AA) into the PNIPAM backbone shifts the LCST of the copolymer from 32 °C up to as high as 60 °C, depending on the amount of incorporated AA [30]. Within that temperature range, PNIPAM-based hydrogels can undergo completely reversible swelling-collapsing volume changes in response to temperature changes in the environment. The temperature-responsive properties of PNIPAM-based hydrogels can be combined with the physical and chemical properties of the gold nanoshells by incorporating the nanoshells into the hydrogel network [31]. The embedded nanoshells absorb/scatter NIR light, which will in turn heat the hydrogel layer, causing the structure to collapse [31]. When the temperature of the polymer exceeds the LCST, the hydrogels can release soluble materials held within the hydrogel matrix by collapsing their polymer network, leading to optically-triggerable drug release [32].

**Scheme 2:**
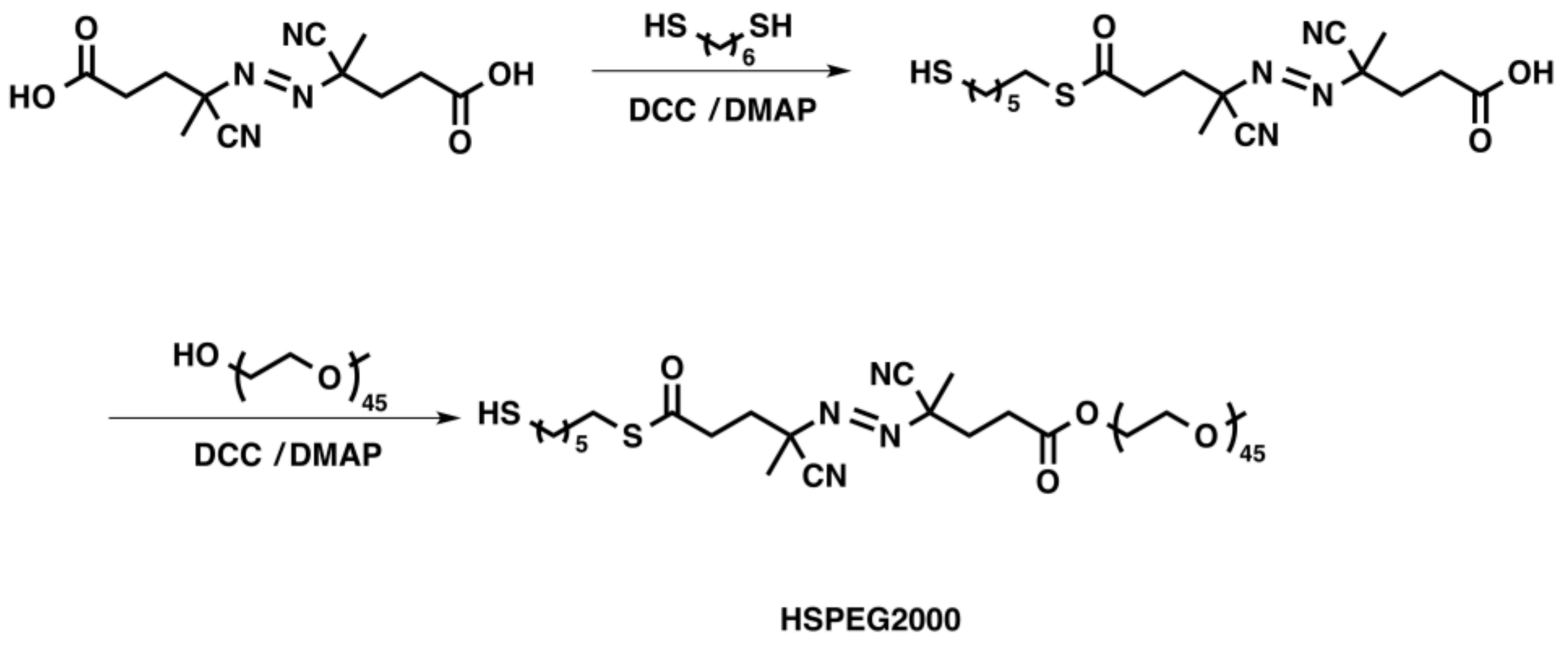
Synthetic strategy used to prepare the free radical initiator **HSPEG2000** [33]. DCC, *N*,*N*′-dicyclohexylcarbodiimide; DMAP, 4-dimethylaminopyridine.

The P(NIPAM-*co*-AA) shell was generated through the free radical polymerization of the rationally-designed initiator **HSPEG2000** (see Scheme 2), synthesized as described previously [33]. The morphology of the resultant hydrogel-coated nanoshells was studied with scanning electron microscopy (SEM), and the temperature-responsive behavior of the particles was characterized by dynamic light scattering (DLS). After the polymer was formed on the surface of the nanoshells, the periphery of the hydrogel layer was further functionalized with avidin, a tetrameric protein that can bind with high affinity to four biotin molecules (K_d_ = 10^−13^~10^−16^ M) [34]. We chose to use the avidin/biotin system in this study for at least three reasons. First, biotin is a naturally-occurring vitamin found in every living cell [35]; specifically, the tissues with the highest amounts of biotin are found in the kidney, pancreas and liver [35]. Cancerous tumors are also known to have more biotin than normal tissue [35]. For drug-delivery applications, the latter feature should act as a driving force for avidin-conjugated particles to reach specific sites with cancerous tissue [35]. Second, a wide variety of biotinylated molecules that can withstand a variety of environments are commercially available. Third, the biotinylated molecules can be readily linked to avidin and, consequently, to the surface of the P(NIPAM-*co*-AA) hydrogel layer via the pendant AA moieties. In the work presented here, the presence of avidin was confirmed by complexing the particles with a fluorescent biotin (biotin-4-FITC). The successful application of this method shows that the hydrogel-coated nanoshells can be modified with essentially any biotinylated target molecule, providing targeted delivery in medical applications. Furthermore, the heat produced from the absorption of NIR light by the nanoparticles can be controlled to promote the collapse of the hydrogel layer and release specific payloads (e.g., drugs) on command.

## 2. Results and Discussion

### 2.1. Characterization of the Gold-Coated Silica Nanoshell Particles

**Figure 1:**
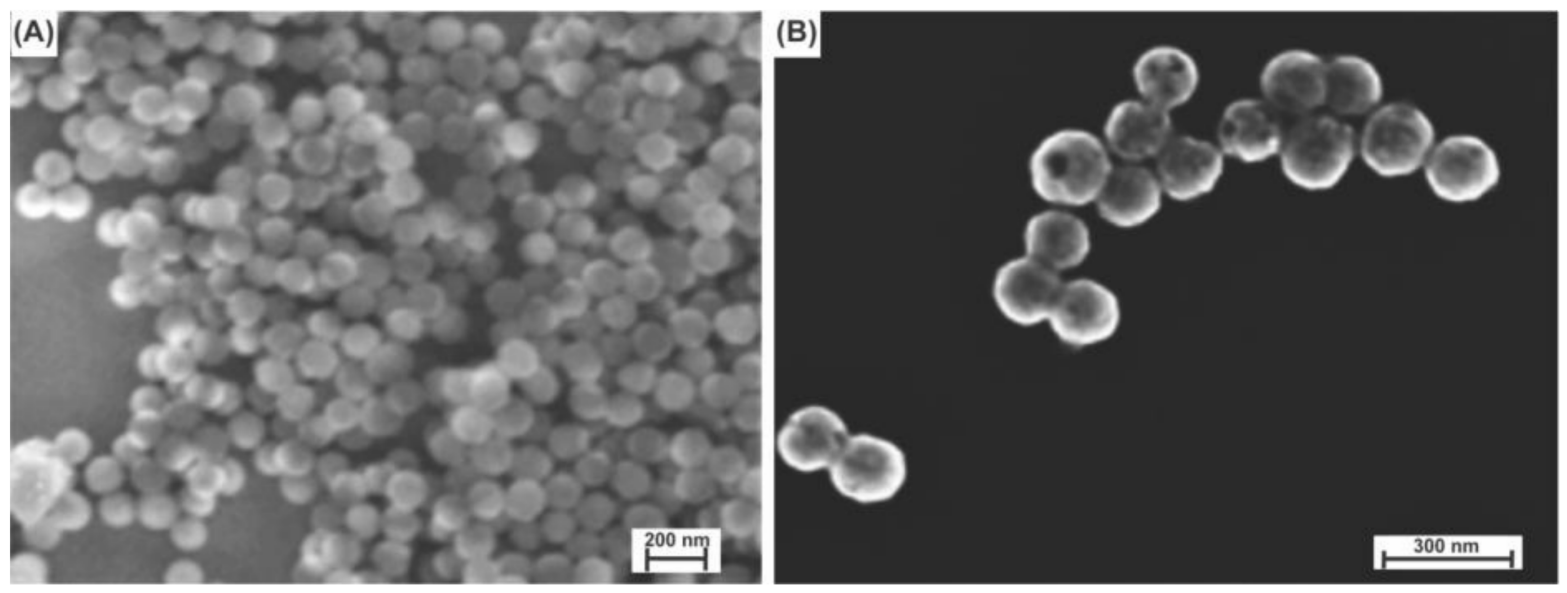
SEM images of (**A**) SiO_2_ cores (~120 nm in diameter) and (**B**) gold nanoshells (~160 nm in diameter). The scale bars are 200 and 300 nm, respectively, where the magnified image on the right illustrates the increase in diameter arising from the gold coating.

Gold nanoshells were successfully fabricated with a ~120 nm silica core and a shell thickness of ~20 nm (see the Experimental Section for the synthetic procedure); representative SEM images of the SiO_2_ nanoparticles and the gold nanoshells are shown in Figure 1A,B, respectively. The diameters of the gold nanoshells are notably larger than those of the solid SiO_2_ nanoparticles. In addition, the surfaces of the nanoshells exhibit topological roughness on the nanometer scale, consistent with that reported previously [13]. The resultant gold nanoshells were then used as templates to grow temperature-responsive P(NIPAM-*co*-AA) hydrogel layers on the surface as described below.

### 2.2. Immobilization of Initiators and Copolymerization of NIPAM and AA on the Surface of the Nanoshells

To grow the hydrogel layer around the gold nanoshells, we first immobilized the free radical initiator **HSPEG2000** on the surface of the nanoshells. The strong sulfur-gold bond of ~50 kcal/mole drives the immobilization of the initiator molecule on the surface of the nanoshell [36]. The sulfur atom of the thiol acts as the headgroup by binding to the gold surface of the nanoshell while exposing the bulky PEG2000 (tailgroup) toward the surrounding solution. The tailgroup of the initiator monolayer also provides steric hindrance between the nanoshell particles; the resulting steric hindrance disrupts the inter-particle interaction, which typically leads to nanoshell aggregation, allowing the nanoshells to be stable in aqueous solution for up to six months under ambient conditions [33]. 

**Figure 2:**
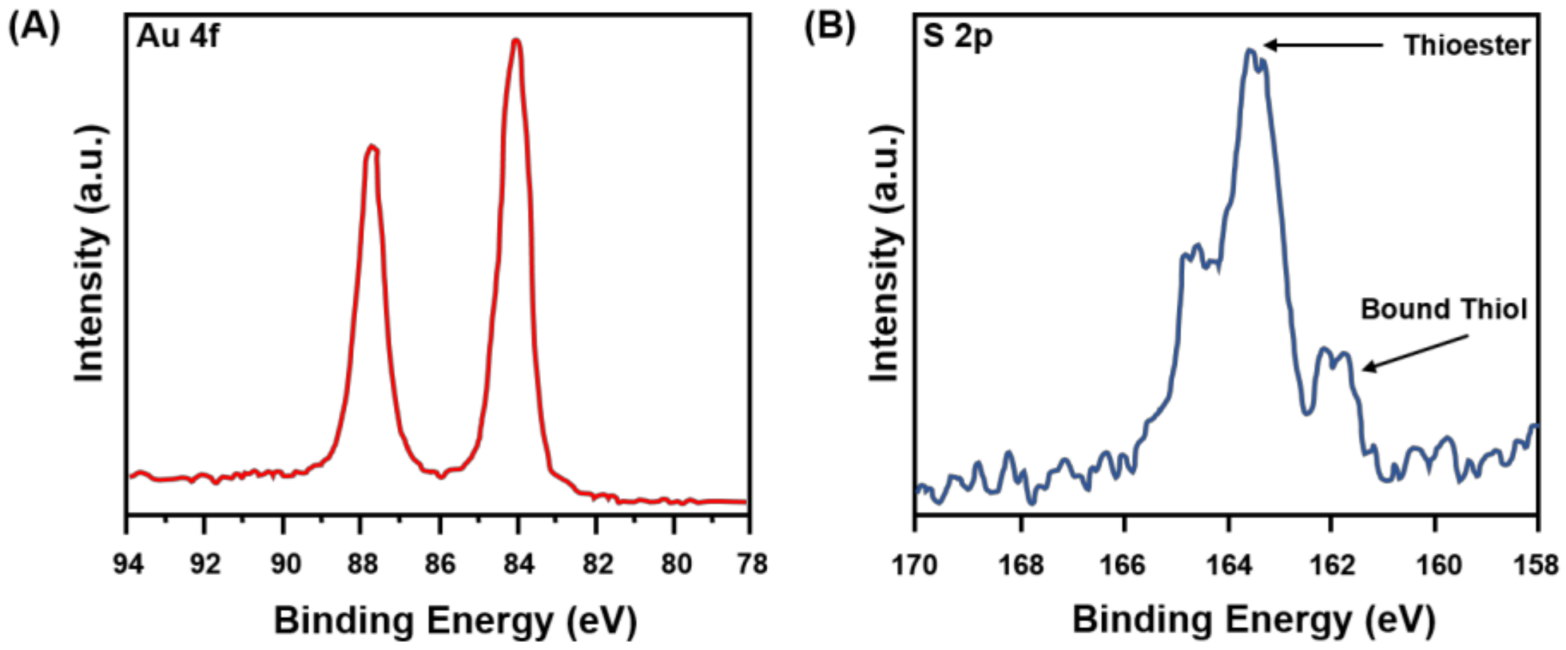
XPS spectra for **HSPEG2000**-functionalized nanoshells for (**A**) the Au 4f region and (**B**) the S 2p region.

After immobilization of **HSPEG2000**, the newly-formed Au-S covalent bond was verified through analysis by X-ray photoelectron spectroscopy (XPS). In analyses by XPS, the binding energy (BE) of core electrons is greatly affected by the oxidation state of the atom of interest and its surroundings [37]. In particular, the S 2p region of the XPS spectra can be used to evaluate the nature of the S atom in self-assembled monolayers (SAMs) on gold [38]. The binding energy will be different for a bound thiolate, an unbound thiolate, and a thioester. However, spin-orbit coupling can inhibit an accurate analysis [38,39]. For example, the binding energy of the S 2p_3/2_ peak for a bound thiol onto an Au surface is 162 eV, whereas the same peak for an unbound thiol appears at ~164 eV in the XPS spectra [38]; at the same time, the peak position for the S 2p_3/2_ of a thioester also appears at ~164 eV [40]. For the S 2p photoelectrons, spin-orbit coupling gives rise to a doublet with an energy difference of 1.2 eV [38]. Figure 2 shows the high-resolution XPS spectra for the Au 4f and S 2p regions of the initiator-functionalized nanoshells. Figure 2A shows the Au 4f region, and Figure 2B shows the S 2p region for the **HSPEG2000**-functionalized nanoshells, confirming the presence of a bound thiolate at 162 eV and a thioester at 164 eV. The S 2p peak for the thioester is more intense than that for the thiol due to attenuation of the thiol photoelectrons by the intervening methylene groups.

Another major focus of concern regarding the functionalized nanoshells is the evaluation of the presence of unbound thiols versus bound ones on the surface of the nanoshells. For example, the S 2p_3/2_ doublet at ~164 eV in Figure 2B can be attributed to either unbound **HSPEG2000** species or to the sulfur of the thioester. We believe this is the latter case given the relative attenuation of the photoelectrons within the **HSPEG2000** initiator and the location of the various sulfur moieties around the nanoshells. The sulfur of the bound thiol is located deeper within the monolayer, closer to the nanoshell-**HSPEG2000** interface, while the sulfur of the thioester is closer to the outer interface. In a layered structure, attenuation tends to underestimate the elements buried deeper (Au-S) relative to those near the outer surface (thioester sulfur), giving a smaller intensity for the buried atoms in the XPS spectrum [40,41]. The attenuation of the bound sulfur can also explain the higher thioester to bound thiol ratio observed in the spectrum compared to the theoretical value of 1:1. Overall, the XPS data show that the initiator molecules were immobilized on the surface of the nanoshells. Due to the close proximity of the BE of an unbound thiol and a thioester, it is possible that the nanoshells have unbound **HSPEG2000**. However, based on the above discussion, we believe that most of the initiator molecules were immobilized on the nanoshell surface.

**Figure 3:**
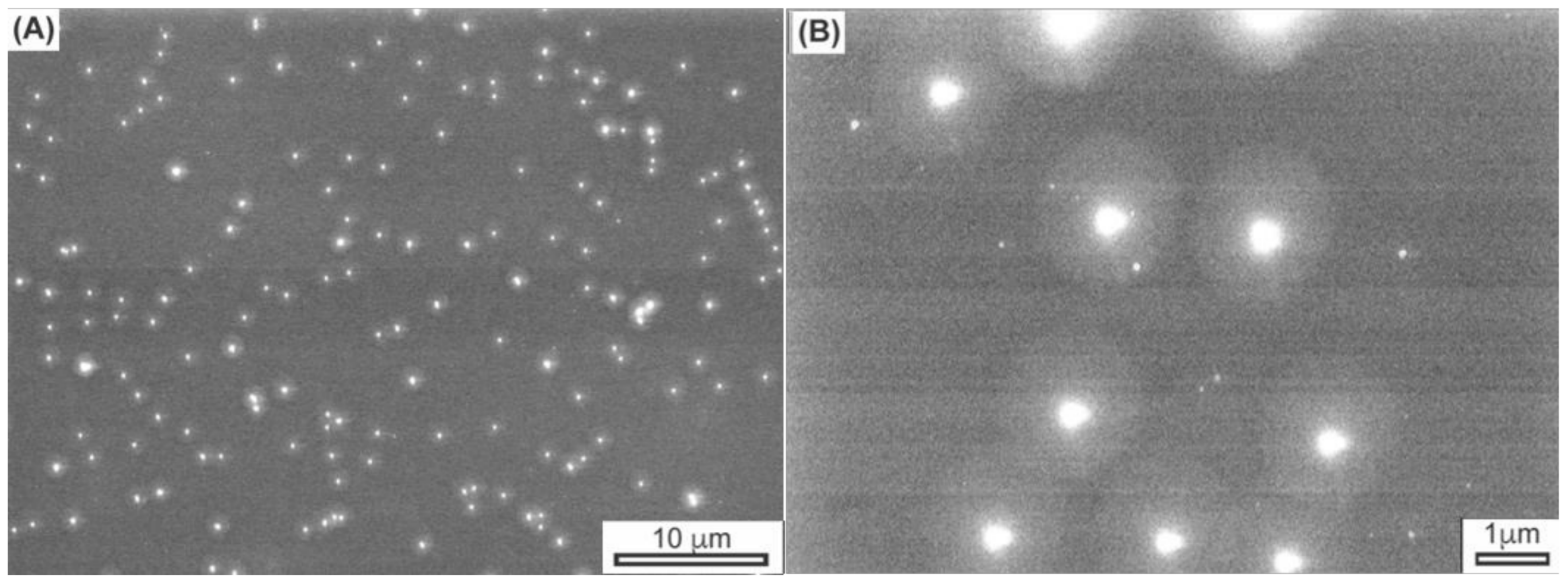
SEM images of (**A**) the hydrogel-coated nanoshells and (**B**) an enlarged-view of the same sample.

Following the immobilization of the initiator molecule, the nanoshells where then encapsulated within the P(NIPAM-*co*-AA) hydrogel by thermally-activating the initiator in the presence of the monomers (see the Experimental Section for details). Figure 3 displays SEM images of the hydrogel-coated nanoshells. The imaged composite consists of a nanoshell core having a diameter of ~160 nm encased by a hydrogel layer; the pale halos surrounding the brighter nanoshell centers provide evidence of the existence of the hydrogel coating on the nanoshells. The SEM images also show that most of the nanoshells were encapsulated with the P(NIPAM-*co*-AA) layers, and more importantly, the nanoshells that have the coating were completely covered, as reflected in the contrast of the SEM images in Figure 3B. We note, however, that these images cannot be used to determine the thickness of the hydrogel coating due to artifacts arising from drying and/or charging; instead, we determined the hydrogel thicknesses (i.e., hydrodynamic dimensions) using dynamic light scattering (DLS) as described below.

### 2.3. Temperature-Responsive Behavior of the Hydrogel-Coated Gold Nanoshells

**Figure 4:**
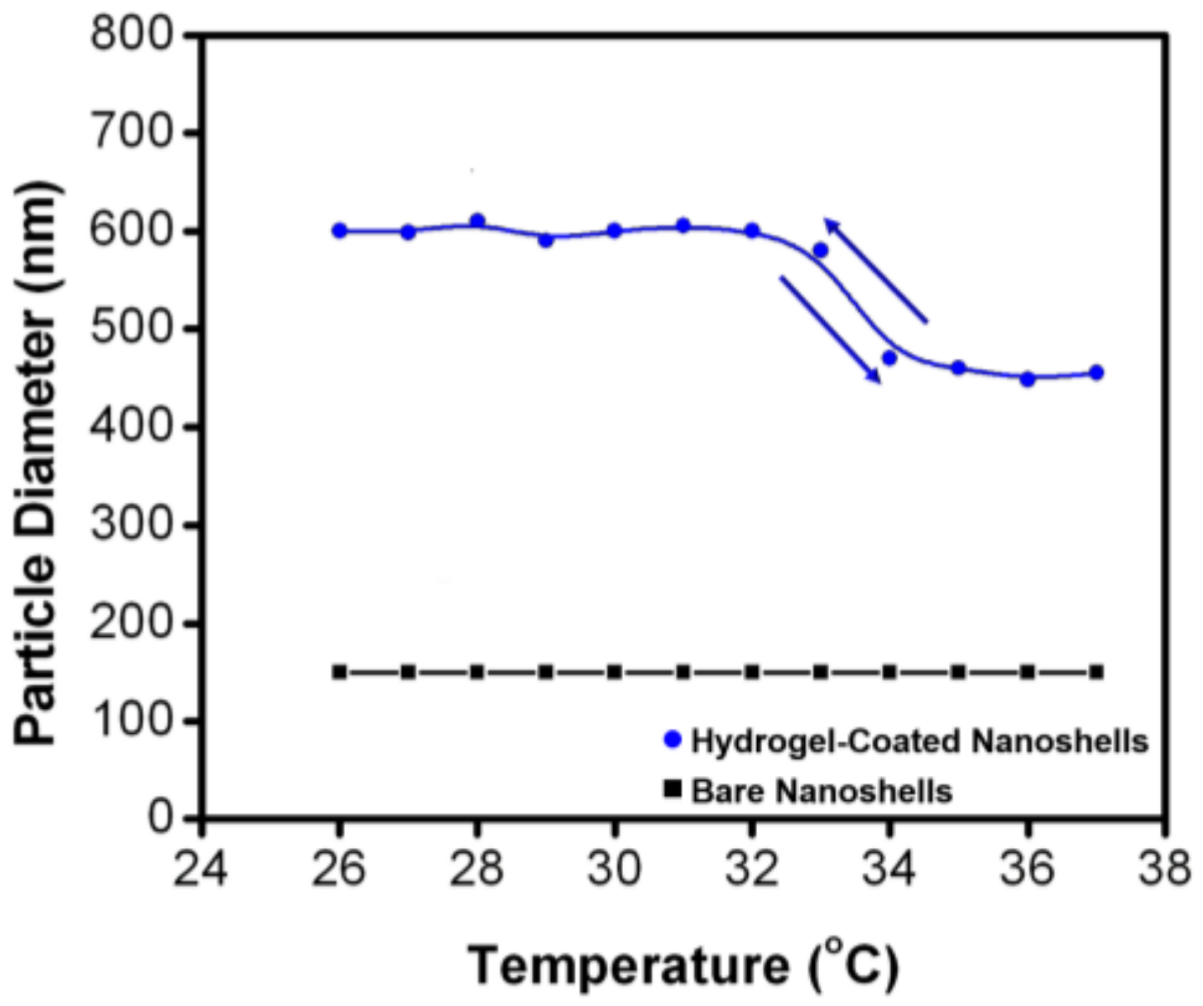
The hydrodynamic diameter of bare nanoshells (black square) and hydrogel-coated nanoshells (blue circle) as a function of temperature. The data shown correspond to a representative set taken from three separate experiments.

The temperature-responsive behavior of the bare gold nanoshells and hydrogel-coated nanoshells was studied by DLS at various temperatures. Figure 4 shows the hydrodynamic diameter of the nanoparticles as a function of solution temperature. The hydrodynamic diameter of the hydrogel-coated nanoshells changed systemically with an increase or decrease in the temperature, between 25 and 40 °C; however, the diameters of the bare nanoshells remained constant under the tested conditions. More specifically, the diameters of the hydrogel-coated nanoshells decreased by ~70 nm upon heating to a temperature of 40 °C, which signified the collapse of the hydrogel structure. Nonetheless, upon cooling to 25 °C, the hydrogel structure swelled back to its original size. The changes in the hydrodynamic diameter of the hydrogel-coated nanoshells were completely reversible with repeated heating and cooling cycles. It should also be noted that the temperature of the phase transition (lower critical solution temperature (LCST)) of our system, containing 5 wt % AA, is 34 °C (see Figure 4). The LCST of our system, determined from three separate temperature profiles including that shown in Figure 4, is about 2 °C higher than the value of a pure NIPAM polymer system and is in good agreement with the results of a previous report [42]. The incorporation of ionizable groups, such as AA or amide groups, into the hydrogel network provides more hydrophilic sites along the polymer backbone, which lead to extensive hydrogen bonding between water and the hydrogel network. By taking into consideration that the collapse of the hydrogel structure above the LCST results from a disruption of the hydrogen bonding between water and the hydrophilic sites of the hydrogel network [43], the higher LCST for our system appears to be reasonable when ionizable groups are incorporated into the polymer backbone. The ability to raise the LCST upon addition of AA monomers presents the possibility of tuning the LCST of the polymer system to be close to the physiological body temperature of humans. Access to a higher LCST renders these P(NIPAM-*co*-AA) hydrogel-coated nanoshells more attractive for potential applications as dynamic materials in the human body.

### 2.4. Optical Properties of the Hydrogel-Coated Gold Nanoshells

**Figure 5:**
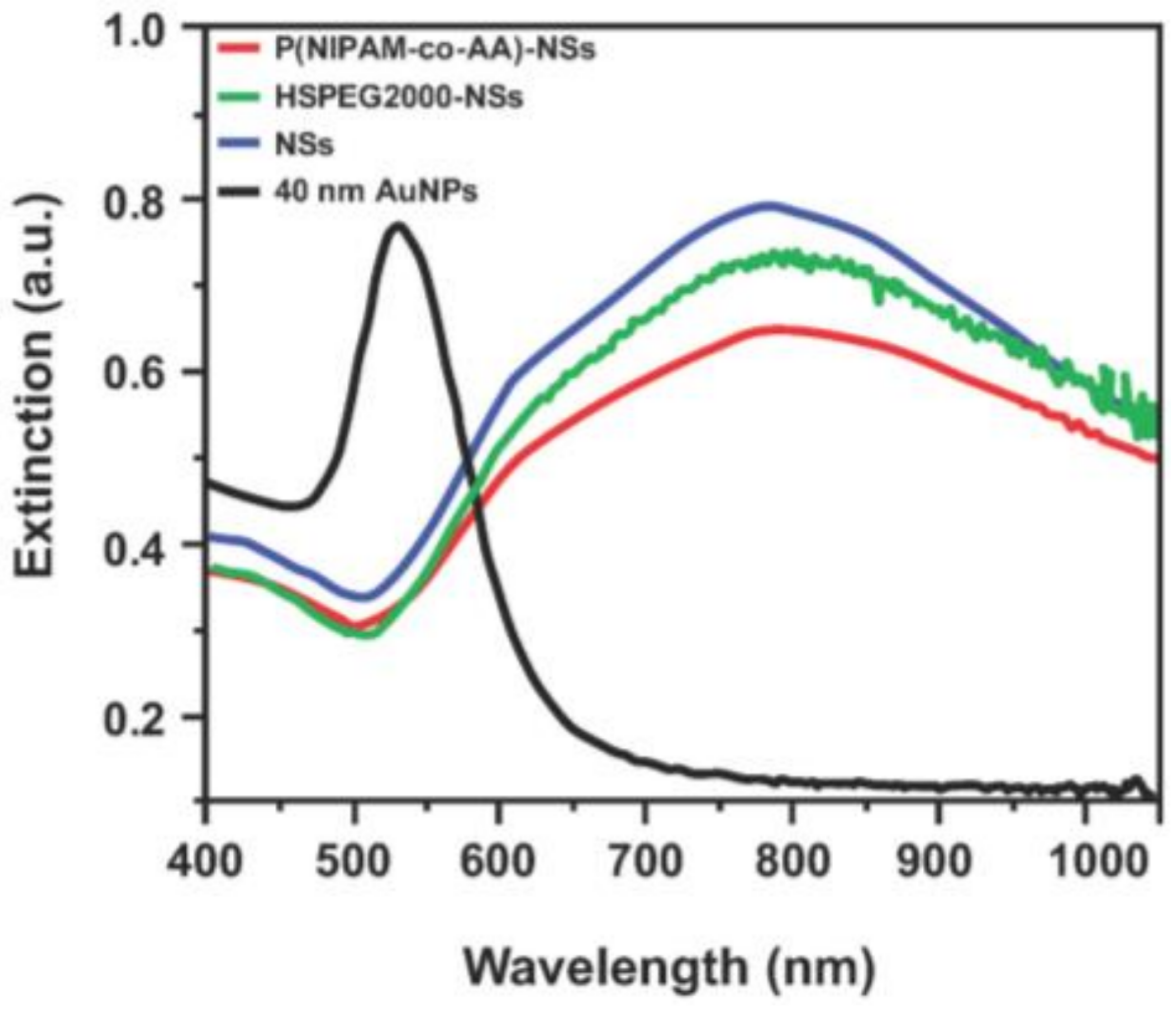
UV-Vis spectra of the 40-nm AuNPs (black), bare nanoshells (blue), **HSPEG2000**-modified nanoshells (green) and the hydrogel-coated nanoshells (red).

The optical properties of 40-nm AuNPs (included for comparison), the bare nanoshells (**NSs**), initiator-functionalized (**HSPEG2000-NSs**), and hydrogel-coated nanoshells (**P(NIPAM-co-AA)-NSs**) were studied by UV-Vis spectroscopy (see Figure 5). For the solid gold nanoparticles, the extinction maximum appears at ~530 nm, which is characteristic of small gold nanoparticles [44]. In contrast, the plasmon resonance of the bare gold nanoshells shifts to a much longer wavelength, showing an extinction maximum at ~800 nm. Previous studies have shown that the plasmon resonance of gold nanoshells can be tuned by varying the size of the silica core or the thickness of the gold shell [14]. For our system, gold nanoshells were fabricated with a ~20 nm-thick gold layer and a ~120 nm-diameter silica core, which led to a strong, broad absorption maximum at ~800 nm. A large red-shift in the plasmon resonance and the SEM images shown in Figure 1 can indirectly support the successful formation of the nanoshell structure. 

The influence of the organic layers (**HSPEG2000** and the hydrogel) on the plasmon resonance of the gold nanoshells was also examined. Despite the fact that the plasmon resonance of a noble metal is sensitive to the medium in contact with the surface of the metal [45,46], the band positions observed for the initiator-functionalized and hydrogel-coated nanoshells were similar to those observed for the bare nanoshells. The optical properties of the temperature-responsive hydrogel-coated nanoshells make them a potential candidate for photothermally-modulated drug delivery; as the light is absorbed by the nanoshells embedded in the hydrogel network, the absorbed light can be converted to heat, resulting in a higher temperature around the nanoshells and the release of encapsulated drugs [32]. Combining the optical properties of the nanoshells and the temperature-responsive behavior of the P(NIPAM-*co*-AA) hydrogel layer could make this new type of system ideal for applications involving nanoscale drug-delivery vehicles. 

### 2.5. Modification of the Hydrogel-Coated Gold Nanoshells with Avidin

To demonstrate the capacity for surface functionalization and the ultimate use in targeted therapeutics, the temperature-responsive hydrogel-coated nanoshells were further conjugated with the protein avidin to create a surface that can bind with high affinity to biotinylated species. The covalent coupling of avidin to the nanoparticle surface was facilitated by activating the carboxyl groups of the P(NIPAM-*co*-AA) hydrogel layer (details are provided in the Experimental Section). The activated carboxyl groups were conjugated to avidin via the ε-amino groups present in the protein’s lysine, which leads to the formation of amide bonds with avidin [47]. After bioconjugation, the modified diameter of the nanoshells increased by ~7 nm, as measured by DLS, which is consistent with the dimensions of an avidin layer, 6.0 nm × 5.5 nm × 4.0 nm, around the hydrogel-coated nanoshells [48]. 

**Figure 6:**
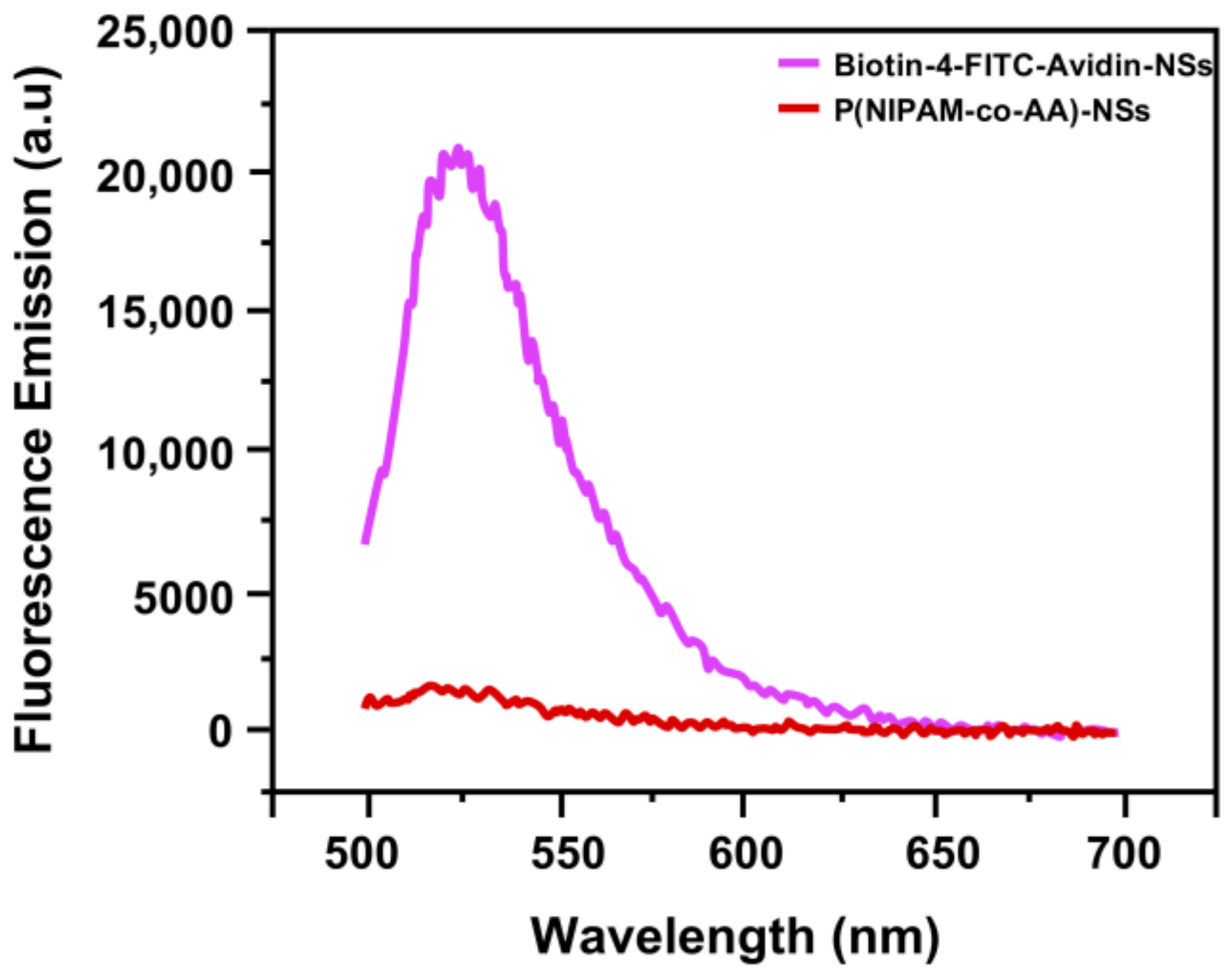
Fluorescence spectra of avidin-nanoparticles complexed with biotin-4-FITC (magenta) and the control experiment (hydrogel nanoshells, red).

In addition to obtaining the diameter of the conjugated nanoshells, we also verified the attachment of avidin by using a fluorescently-tagged biotin (biotin-4-FITC). The hydrogel-coated nanoshells were mixed with biotin-4-FITC dissolved in a buffer solution followed by three washing steps after the reaction to ensure the removal of unbound biotin-4-FITC from the solution. As a control experiment, hydrogel-coated nanoshells, which were not modified with avidin molecules, were mixed with biotin-4-FITC using the same procedure. The fluorescence spectra for the avidin-nanoshells complexed with biotin-4-FITC and the control experiment are shown in Figure 6. For the avidin-nanoshells, the emission maximum occurs at 524 nm, providing a similar spectrum to that of biotin-4-FITC dissolved in PBS buffer. Although gold nanoparticles are known to serve as ultra-efficient quenchers of the molecular excitation energy in a chromophore via their surface-energy-transfer properties [49], the fluorescence properties of the biotin-4-FITC were still in a detectable range, probably due to the presence of the thick hydrogel layer around the gold nanoshells. In contrast, for the control experiment, almost no fluorescence properties were detected, indicating that all the biotin-4-FITC molecules were washed out during the washing process.

**Figure 7:**
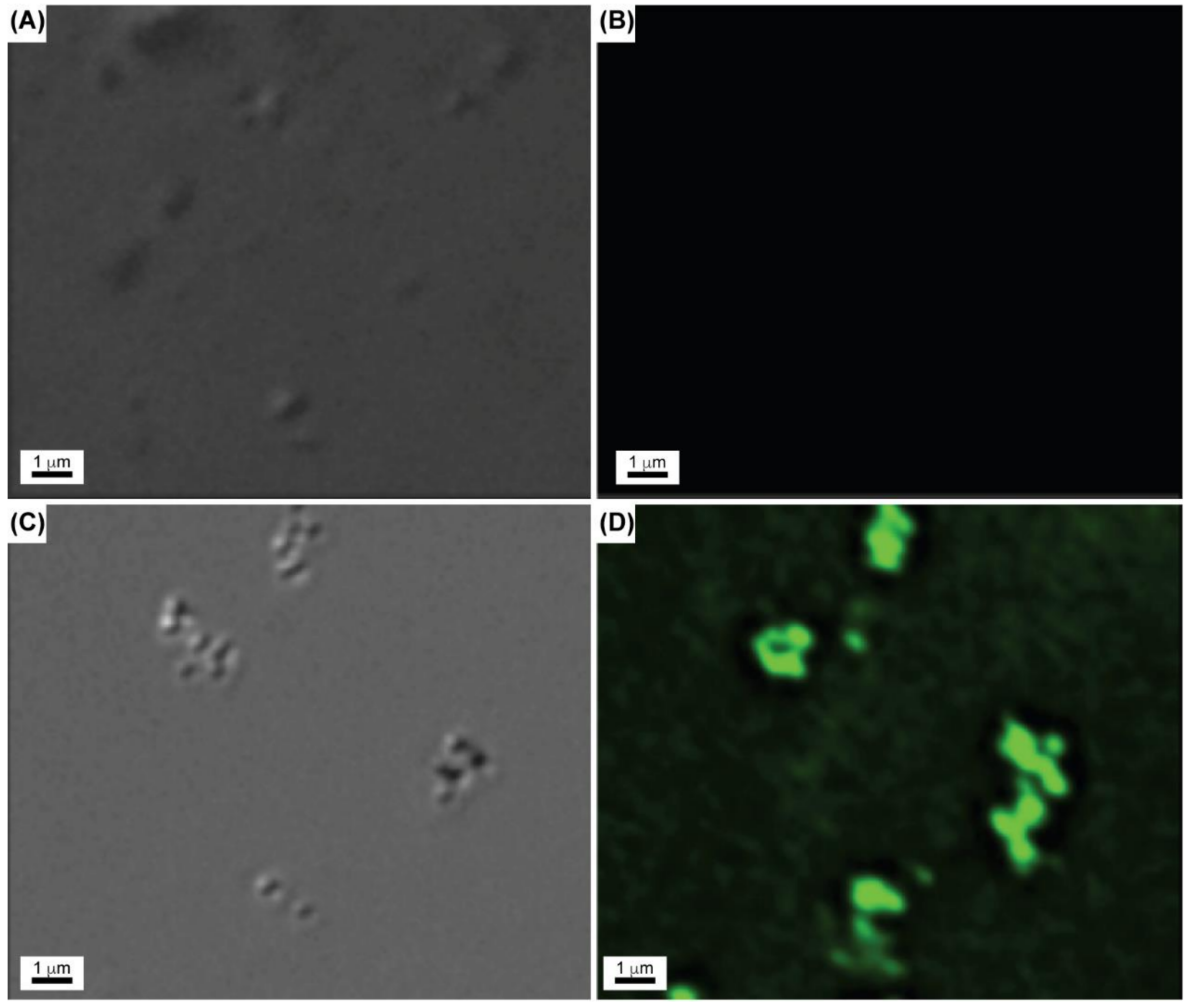
Confocal microscope images of: (**A**) control experiment in normal brightfield mode; (**B**) control experiment in fluorescence mode; (**C**) avidin-nanoparticles complexed with biotin-4-FITC in normal brightfield mode; and (**D**) avidin-nanoparticles complexed with biotin-4-FITC in fluorescence mode.

To support this observation, the conjugated nanoshells suspended in PBS were visualized by confocal microscopy in normal brightfield and fluorescence modes. Figure 7 shows the brightfield and fluorescence confocal images for avidin-nanoshells complexed with biotin-4-FITC along with images for the control experiment. Fluorescence was only observed for the avidin-nanoshells complexed with biotin-4-FITC, in agreement with the fluorescence spectroscopy. From these experiments, we can conclude that avidin was successfully conjugated to the surface of the hydrogel-coated nanoshells, providing potential utilization of the immobilized avidin to attach various types of biotinylated target molecules around the hydrogel-coated gold nanoshells. Additionally, the results also provide evidence that the avidin molecules on the nanoshell surface remain active, as demonstrated by the ability to bind biotin even after conjugation. 

In previous studies [31,32,50,51,52], we demonstrated the potential of various hydrogel-coated gold nanoparticles as vehicles for loading and thermally-triggered payload release by illustrating their dynamic behavior in response to systematic changes in temperature. Chemically cross-linked PNIPAM-*co*-AA hydrogel particles have a porous network structure, which is particularly suitable to trap small molecules [53]. The advantages of the system reported here lie not only in the capacity for activation by NIR modulation as described above, but also in the covalent attachment of the hydrogels to the nanoshell cores and the ability to grow relatively thick hydrogel overlayers for optimal drug loading and delivery.

## 3. Conclusions

Temperature-responsive hydrogel-coated gold nanoshells were prepared using a rationally-designed surface-bound initiator. The gold nanoshells used for this study were ~160 nm in diameter, and the thickness of the P(NIPAM-*co*-AA) hydrogel was ~200 nm, as characterized by SEM and DLS. The temperature-responsive behavior of the hydrogel-coated nanoshells was demonstrated with DLS, showing a decrease in the particle diameter with increasing solution temperature. The surface plasmon resonance of the hydrogel-coated nanoshells appeared at ~800 nm, which is a particularly important spectral range for further biomedical applications. Furthermore, the periphery of the hydrogel-coated nanoshells was conjugated with the model protein avidin. The successful bioconjugation of the nanoparticles indicates that coupling of biotinylated targeting moieties and other biomolecules (e.g., proteins, DNA, and antibodies) is possible with this system. Combining all of the highly functionalized properties, our temperature-responsive hydrogel-coated gold nanoshells offer considerable promise for use in various biotechnological applications.

## 4. Materials and Methods

### 4.1. Materials

All reagents were purchased from the indicated suppliers and used without further purification unless indicated otherwise: ammonium hydroxide (30% NH_3_), tetraethyl orthosilicate (TEOS, 99%), 3-aminopropyltrimethoxysilane (3-APTMS, 97%), tetrakis(hydroxymethyl)phosphonium chloride (THPC, 80% in H_2_O), potassium carbonate (K_2_CO_3_, 99%), 4,4′-azobis(4-cyanovaleric acid) (75%), 1,6-hexanedithiol (96%), *N*-(3-dimethylaminopropyl)-*N*′-ethylcarbodiimide hydrochloride (EDC, 98%), poly(ethylene glycol) methyl ether (Mn 2000) and avidin from egg white (avidin) (Aldrich, St. Louis, MO, USA); sodium hydroxide (NaOH, 98%) and formaldehyde (HCOH, 37%) (EM Sciences, Hatfield, PA, USA); *N*-isopropylacrylamide (NIPAM, 99%), acrylic acid (AA, 99.5%), *N*,*N*′-methylenebisacrylamide (BIS, 96.0%); 4-dimethylaminopyridine (DMAP, 99.0%) (Acros); *N*,*N*′-dicyclohexylcarbodiimide (DCC, Fluka, 99.0%); absolute ethanol (McCormick Distilling Co., Weston, MO, USA); hydrogen tetrachloroaurate (HAuCl_4_, Strem, Newburyport, MA, USA); biotin-4-fluorescein (biotin-4-FITC, >99%, Biotum, Fremont, CA, USA); tetrahydrofuran, hexane, ethyl acetate, chloroform, dichloromethane and methanol (Avantor, Center Valley, PA, USA). NIPAM was recrystallized in hexane and dried under vacuum before use. Tetrahydrofuran was freshly distilled over calcium hydride (Sigma Aldrich, St. Louis, MO, USA) and collected immediately prior to use. All water used in the reactions was purified to a resistance of 10 MΩ (Milli-Q Reagent Water System, Millipore Corporation, Burlington, MA, USA) and filtered through a 0.2 μm filter to remove any particulate matter.

### 4.2. Preparation of Gold Nanoshells

#### 4.2.1. Preparation of Gold-Seeded SiO_2_ Nanoparticles

Silica nanoparticles with a diameter of ~120 nm were prepared by the Stöber method [15]. In order to functionalize the surface of the SiO_2_ particles with -NH_2_ groups, the method by Waddell et al. [54] was followed by adding an excess amount of APTMS (~50 μL) to 100 mL of the SiO_2_ nanoparticle solution. The mixture was allowed to stir for 24 h at room temperature and then refluxed at 80 °C for 1 h. The resulting amine-functionalized SiO_2_ nanoparticles were isolated by centrifugation at 2500 rpm for 1 h and redispersed in absolute ethanol. 

To attach colloidal gold to the amino-functionalized SiO_2_ particles, a method described by Duff et al. [55] was followed. Briefly, 0.5 mL of a 1 M NaOH solution and 1 mL of a THPC solution (prepared by adding 12 μL of 80% THPC in H_2_O to 1 mL of Milli-Q water) were added to 45 mL of Milli-Q water. The mixture was stirred for 5 min after which 2 mL of 1 wt % HAuCl_4_ in water was added to the solution. The mixture was then stirred for 30 min and stored in the refrigerator for at least 3 days before use. Afterwards, 2 mL of the APTMS-coated SiO_2_ nanoparticles solution and 25 mL of concentrated THPC-gold solution were mixed overnight. The gold-seeded SiO_2_ nanoparticles were isolated by centrifugation and redispersed in Milli-Q water (100 mL). 

#### 4.2.2. Nanoshell Growth

To grow the gold layer on the THPC-gold seeded SiO_2_ nanoparticles, we prepared a solution containing a reducible gold salt (K-gold solution). To prepare the K-gold solution, 0.025 g of K_2_CO_3_ were dissolved in 100 mL of Milli-Q water. Afterwards, 2 mL of 1 wt % HAuCl_4_ solution were added. The K-gold solution was stirred at room temperature for 1 h and stored in the refrigerator overnight before use. Gold shells were grown by adding 2 mL of the THPC-gold-seeded SiO_2_ nanoparticles to 40 mL of the K-gold solution. After the reaction mixture was stirred for 10 min, 0.2 mL of formaldehyde were added to the solution to reduce the K-gold. The gold nanoshells (~160 nm in diameter) were centrifuged at 2300 rpm and redispersed in Milli-Q water before use.

### 4.3. Functionalization of the Gold-Nanoshells with P(NIPAM-co-AA)

To functionalize the gold nanoshells with the initiator molecule, 20 mL of the gold nanoshell solution was mixed with 2 mL of a 1 mM solution of **HSPEG2000** (in ethanol) for 30 min and allowed to stand at room temperature for 24 h. The initiator-functionalized nanoshells were washed by centrifugation at 2300 rpm with ethanol and water (each twice) before use. The hydrogel-coated nanoshells were prepared by free radical polymerization in aqueous solution by the following procedure: 10 mL of the initiator-functionalized nanoshells were dispersed in 20 mL of water followed by the addition of 2 mL of NIPAM (0.01 M; 0.20 mmol), 0.2 mL of AA (0.01 M; 0.02 mmol) and 0.2 mL of BIS (0.01 M; 0.02 mmol) [44,56,57]. The solution was stirred and bubbled with argon for 1 h to remove oxygen, which can intercept radicals. To initiate the polymerization, the solution was heated to 65 °C in an oil bath and stirred for 6 h under argon. At the end of the reaction time, the solution was cooled to 20 °C. The hydrogel-coated particles were purified by dialysis (Spectra/Por Dialysis Membrane, MWCO 12–14000, VWR, Radnor, PA, USA) over the course of one week at room temperature; the water used for dialysis was changed daily.

### 4.4. Modification of the Hydrogel-Coated Nanoshells with Avidin and Biotin-4-FITC

The protein avidin was immobilized on the hydrogel layer by linking it to the carboxyl group present on the hydrogel layer. The covalent coupling of avidin to the nanoparticle surface was facilitated by the crosslinker EDC by activating the carboxyl groups of the P(NIPAM-*co*-AA) hydrogel layer. The activated carboxyl groups were conjugated to avidin via the ε-amino groups of the protein’s lysine, which leads to the formation of an amide bond with the avidin. The reaction procedure required 1 mg of DMAP and 2 mg of EDC to be dissolved in 15 mL of the hydrogel-coated nanoshell solution. The avidin, dissolved in 0.25 mL of PBS buffer (0.01 M, pH 7.2), was then added to the nanoshell solution, and the solution was stirred overnight to ensure the amide bond formation between the hydrogel layer and the avidin.

To verify the attachment of avidin to the hydrogel layer, we also performed a study utilizing biotin linked to a fluorescent organic dye (biotin-4-FITC). Specifically, 1 mg of biotin-4-FITC was dissolved in a mixture of 0.5 mL of PBS buffer (0.01M, pH 7.2) and 0.5 mL of a 0.1 M NaOH solution. Thirty microliters of the biotin solution were added to 7 mL of the avidin-conjugated nanoshell solution while stirring. As a control experiment, the same amount of biotin solution was also added to the nanoshell solution, which was not conjugated with the avidin molecule. The solutions were stirred for 30 min and washed by centrifugation three times at 5000 rpm to remove the unbound biotin-4-FITC and stored in the refrigerator until use.

### 4.5. Characterization Methods

To characterize the SiO_2_ nanoparticles, bare gold nanoshells, initiator-functionalized nanoshells, and hydrogel-coated nanoshells X-ray photoelectron spectroscopy (XPS), scanning electron microscopy (SEM) dynamic light scattering (DLS) and ultraviolet-visible (UV-Vis) spectroscopy were used. Avidin-biotin conjugated nanoparticles were characterized by fluorescence spectroscopy and confocal microscopy.

#### 4.5.1. X-ray Photoelectron Spectroscopy

XPS spectra of the initiator-functionalized nanoshells were collected using a PHI 5700 X-ray photoelectron spectrometer (Physical Electronics, Chanhassen, MN, USA) equipped with a monochromatic Al Kα X-ray source (hυ = 1486.7 eV) incident at 90° relative to the axis of a hemispherical energy analyzer. The initiator-functionalized nanoshells were deposited onto a silicon wafer, and the solvent was allowed to evaporate before analysis. The spectrometer was operated at high resolution with a pass energy of 23.5 eV, a photoelectron takeoff angle of 45° from the surface and an analyzer spot diameter of 2 nm. The base pressure in the chamber during the measurements was 3 × 10^−9^ Torr, and the spectra were collected at rt. After collecting the data, the binding energies of the S and C peaks were referenced by setting the Au 4f_7/2_ binding energy to 84 eV. 

#### 4.5.2. Scanning Electron Microscopy

Analysis by SEM was performed using a LEO-1525 Scanning Electron Microscope (Carl Zeiss AG, Thorwood, NY, USA) with 20 kV of accelerating voltage and a JEOL JSM 6400 Scanning Electron Microscope (JEOL, Peabody, MA, USA) with 10 kV of accelerating voltage during the measurements. Bare SiO_2_ nanoparticles, gold-silica nanoshells and hydrogel-coated nanoshells were deposited on silicon wafers and dried at room temperature to collect the images. The overall morphology of the particles was examined with SEM.

#### 4.5.3. Dynamic Light Scattering

For the DLS measurements, an ALV-5000 Multiple Tau Digital Correlation instrument (ALV-Laser Vertriebsgesellschaft mbH, Langen, Hesse, Germany) was used, operating at a light source wavelength of 514.5 nm and a fixed scattering angle of 90°. The hydrodynamic diameters of the bare nanoshells and the hydrogel-coated nanoshells were measured as a function of temperature in water. The samples were analyzed at dilute concentrations, and all of the collected data showed good Gaussian distribution curves. 

#### 4.5.4. UV-Vis Spectroscopy

The optical properties of the 40 nm AuNPs, initiator-functionalized nanoshells and the hydrogel-coated nanoshells were monitored at room temperature using a Cary 50 Scan UV-Vis optical spectrometer (Varian, Palo Alto, CA, USA) in conjunction with Cary Win UV software (Varian, Palo Alto, CA, USA). UV-Vis spectra of the prepared nanoparticles were collected in solution over a wavelength range of 400–1100 nm in a quartz cuvette having a 1 cm optical path length. 

#### 4.5.5. Fluorescence Measurements

For the fluorescence study, the fluorescence spectra of the avidin-nanoparticles complexed with biotin-4-FITC and the control experiment were measured by a PTI (Photon Technology International) Xe lamp and steady-state fluorescence (Photon Technology International, Birmingham, NJ, USA). The collected data were analyzed by the software FeliX32 Analysis (Version 1.2, Photon Technology International, Birmingham, NJ, USA). The particles were also visualized by a Leica TCS SP2 confocal microscope (Leica, Mannheim, Germany) equipped with 488 nm argon and 543 nm HeNe lasers in normal brightfield mode and fluorescence mode.
